# Longitudinal changes in the nasopharyngeal resistome of South African infants using shotgun metagenomic sequencing

**DOI:** 10.1371/journal.pone.0231887

**Published:** 2020-04-22

**Authors:** Rendani I. Manenzhe, Felix S. Dube, Meredith Wright, Katie Lennard, Heather J. Zar, Stephanie Mounaud, William C. Nierman, Mark P. Nicol, Clinton Moodley

**Affiliations:** 1 Division of Medical Microbiology, Faculty of Health Science, University of Cape Town, Cape Town, South Africa; 2 Department of Molecular and Cell Biology, Faculty of Science, University of Cape Town, Cape Town, South Africa; 3 J. Craig Venter Institute, Rockville, MD, United States of America; 4 Division of Computational Biology, Faculty of Health Sciences, University of Cape Town, Cape Town, South Africa; 5 Department of Paediatrics and Child Health, Red Cross War Memorial Children’s Hospital and MRC unit on Child & Adolescent Health, University of Cape Town, Cape Town, South Africa; 6 Division of Infection and Immunity, University of Western Australia, Perth, Australia; 7 National Health Laboratory Service, Groote Schuur Hospital, Cape Town, South Africa; Universidad Nacional de la Plata, ARGENTINA

## Abstract

**Introduction:**

Nasopharyngeal (NP) colonization with antimicrobial-resistant bacteria is a global public health concern. Antimicrobial-resistance (AMR) genes carried by the resident NP microbiota may serve as a reservoir for transfer of resistance elements to opportunistic pathogens. Little is known about the NP antibiotic resistome. This study longitudinally investigated the composition of the NP antibiotic resistome in *Streptococcus*-enriched samples in a South African birth cohort.

**Methods:**

As a proof of concept study, 196 longitudinal NP samples were retrieved from a subset of 23 infants enrolled as part of broader birth cohort study. These were selected on the basis of changes in serotype and antibiogram over time. NP samples underwent short-term enrichment for streptococci prior to total nucleic acid extraction and whole metagenome shotgun sequencing (WMGS). Reads were assembled and aligned to pneumococcal reference genomes for the extraction of streptococcal and non-streptococcal bacterial reads. Contigs were aligned to the Antibiotic Resistance Gene-ANNOTation database of acquired AMR genes.

**Results:**

AMR genes were detected in 64% (125/196) of the samples. A total of 329 AMR genes were detected, including 36 non-redundant genes, ranging from 1 to 14 genes per sample. The predominant AMR genes detected encoded resistance mechanisms to beta-lactam (52%, 172/329), macrolide-lincosamide-streptogramin (17%, 56/329), and tetracycline antibiotics (12%, 38/329). *Msr*D, *erm*B, and *mef*A genes were only detected from streptococcal reads. The predominant genes detected from non- streptococcal reads included *bla*_OXA-60_, *bla*_OXA-22_, and *bla*_BRO-1_. Different patterns of carriage of AMR genes were observed, with only one infant having a stable carriage of *mef*A, *msr*D and *tet*M over a long period.

**Conclusion:**

This study demonstrates that WMGS can provide a broad snapshot of the NP resistome and has the potential to provide a comprehensive assessment of resistance elements present in this niche.

## Introduction

Infection with antibiotic-resistant bacteria is a major public health concern due to the limited availability of new treatment options [1]. Increasing antibiotic resistance has been noted in respiratory tract bacterial pathogens which are capable of causing life-threatening infections [2,3]. The upper respiratory tract, including the nasopharynx, is the reservoir for many respiratory pathogens and may also serve as a source for the transfer of antimicrobial-resistance (AMR) genes from non-pathogenic to pathogenic bacteria [4].

Pathogens which commonly colonize the upper airways include *Streptococcus pneumoniae* (the pneumococcus), *Staphylococcus aureus*, *Haemophilus influenzae*, *Neisseria meningitidis*, and several Gram-negative bacilli [5–7]. The pneumococcus and *H*. *influenzae* are among the leading causes of bacterial respiratory tract infections in young children [8,9]. Asymptomatic NP carriage of pneumococci is prevalent among infants and often precedes the development of disease [4,10]. Drug-resistant pneumococci may cause difficult-to-treat infections, associated with increased morbidity and mortality [4,10]. In many cases, antibiotic resistance results from horizontal gene transfer (HGT) of a mobile genetic element, or uptake of free DNA from the surrounding environment [11,12], which is of particular importance as pneumococci are naturally competent [13].

Culture-based methods only allow for the detection of certain AMR genes in viable, culturable bacteria, and are therefore unable to completely characterise the resistome in a particular niche [14]. An alternative approach for detection of AMR genes is whole metagenome shotgun sequencing (WMGS) of DNA extracted directly from samples [15,16]. The majority of WMGS studies of the antibiotic resistome have focused on the human gut resistome [16–20]. To our knowledge there are no published studies of the NP antibiotic resistome.

We have previously reported changes in NP pneumococcal antibiotic-resistance in infants studied longitudinally over the first year of life, using culture-based susceptibility testing [21]. Here we further characterise a subset of these samples using WMGS to demonstrate proof of concept for resistome analysis of upper respiratory tract samples.

## Material and methods

This study was nested within a birth-cohort study which investigates the within-host micro-evolution of naturally acquired pneumococci in 800 infants. NP swabs were longitudinally collected fortnightly from birth until twelve months, in this high-carriage African setting [22]. As a proof of concept, we selected obtained 196 NP swabs from 23 infants, on the basis of longitudinal changes in serotype and antibiogram over the first year of life, for shotgun metagenomic sequencing [21]. These NP swabs were stored in 1 ml skim milk-tryptone-glucose-glycerol (STGG) medium as previously described [23]. The study was approved by the Faculty of Health Sciences Human Research Ethics Committee of the University of Cape Town (reference numbers: 235/2016 and 401/2009) and written informed consent obtained from all parents or legal guardians at enrolment.

The NP-STGG samples were enriched as previously described, with minor modifications [24]. Briefly, 200 μl of an NP-STGG sample was transferred to 6 ml Todd-Hewitt Broth (without antibiotics), containing 0.5% yeast extract and 17% fetal bovine serum. The broth was incubated at 37°C with 5% CO_2,_ without shaking for 6 hours, then centrifuged at 9000 rpm for 10 minutes at 4°C. Total nucleic acid extraction was performed on the collected pellet using the QIAsymphony SP automated platform (Qiagen, Hilden, Germany) with the QIAsymphony Virus/Bacteria Mini Kit (Cat. No. 931036) following the manufacturer’s instructions. Nucleic acid concentrations and purity were determined by UV spectrophotometry using the NanoDrop® ND-100 (Thermo Fishers Scientific, Waltham, USA).

Total nucleic acid was subjected to shotgun sequencing on the MiSeq platform using the MiSeq Reagent Kit v3 (600-cycle) (Illumina, San Diego, USA) at the J. Craig Venter Institute, Rockville, USA. Metagenomic DNA sequencing and assembly protocols have previously been described [24]. Reads were assembled using metaSPAdes [25], and aligned to a database containing *Streptococcus pneumoniae* complete genomes in order to re-construct the pneumococcal genomes and extract all the streptococcal contigs (genes identified using pneumococcal references may have come from other streptococcus species due to high level of genetic relatedness thus referring to these as streptococcal contigs). Bacterial contigs not mapping to pneumococcal genomes were regarded as non-streptococcal contigs and were separately extracted for further analysis [24].

Screening for AMR genes present in the selected NP samples was performed on the assembled contigs for both streptococcal and non- streptococcal contig datasets. Contigs were aligned to the Antibiotic Resistance Gene-ANNOTation (ARG-ANNOT) database of acquired AMR genes. To assess the reproducibility of the resistome analysis, all bacterial contigs as well as the streptococcal and non-streptococcal contigs were separately aligned against the ARG-ANNOT database. To increase sensitivity for identifying novel genes or genotypes with low levels of similarity to the reference genes, less stringent criteria were used [26]. A sequence with ≥ 90% identity [27], with an alignment coverage length of ≥ 25% to the reference gene sequence was designated as an AMR gene [26]. The AMR genes were manually confirmed.

Statistical analyses were performed using STATA (Stata Corporation, College Station, TX). Chi-square and Fisher’s exact tests were used to compare the differences in the proportion of samples with AMR genes. A *p*-value of <0.05 was considered statistically significant.

## Results

### Participants and metagenomic sample characteristics

A total of 196 longitudinal NP samples were selected from 23 infants, with an average of 9 selected NP samples per infant (range, 4–21 samples). The age at which the NP samples were collected spanned the first year of life with an average age of 15 weeks. Four of the 23 infants were born via caesarean section (Table 1). Eight infants were born to HIV infected mothers, but none of the infants were infected. Antibiotics were administered to 6 out of 7 infants who had severe or non-severe lower respiratory tract infection (LRTI) during the first year of life (ages, 0–52 weeks) (Table 1).

**Table 1:** Clinical characteristics of the participants.

Infant no.	Mode of delivery	HIV exposed	LRTI case, age (weeks)	Age antibiotic(s) given (weeks)	Antibiotic for LRTI treatment (days)	Admission for suspected LRTI
1	Normal vaginal	No	0	0	Erythromycin-PO (7 days)	-
					Amikacin-IV (5 days)	
					Cefotaxime-IV (4 days)	
					Gentamycin-IV (3 days)	
					Meropenem-IV (3 days)	
2	Normal vaginal	No	8	8	Amoxicillin-PO (5 days)	-
3	Normal vaginal	No	-	-	-	-
4	Normal vaginal	No	-	-	-	Ambulatory
5	Emergency Caesarean section	No	-	-	-	Ambulatory
6	Normal vaginal	Yes	-	-	-	Both
7	Normal vaginal	Yes	-	-	-	Unknown
8	Elective Caesarean section	No	-	-	-	-
9	Normal vaginal	No	-	-	-	-
10	Normal vaginal	No	25	28	Amoxicillin-PO	Ambulatory
11	Normal vaginal	Yes	-	-	-	Ambulatory
12	Normal vaginal	Yes	8	-	none	Ambulatory
13	Normal vaginal	Yes	-	-	-	-
14	Normal vaginal	No	-	-	-	Ambulatory
15	Normal vaginal	No	-	-	-	-
16	Emergency Caesarean section	Yes	52	52	Amoxicillin-PO (5 days)	Both
					Ampicillin-IV (1 day)	
					Gentamycin-IV (1 day)	
17	Normal vaginal	Yes	36	36	Amoxicillin-PO (8 days)	Ambulatory
					Ampicillin-IV (1 day)	
					Gentamycin-IV (1 day)	
18	Normal vaginal	No	-	-	-	Ambulatory
19	Normal vaginal	No	-	-	-	Ambulatory
20	Normal vaginal	Yes	-	-	-	Hospitalized
21	Normal vaginal	No	-	-	-	Ambulatory
22	Normal vaginal	No	24	24	Amoxicillin-PO (5 days)	Hospitalized
23	Elective Caesarean section	No	-	-	-	Ambulatory

LRTI- Lower respiratory tract infection, PO- Oral antibiotic, IV- Intravenous antibiotic, Both- Ambulatory and acute care.

### Nasopharyngeal resistome characteristics

**Fig 1:**
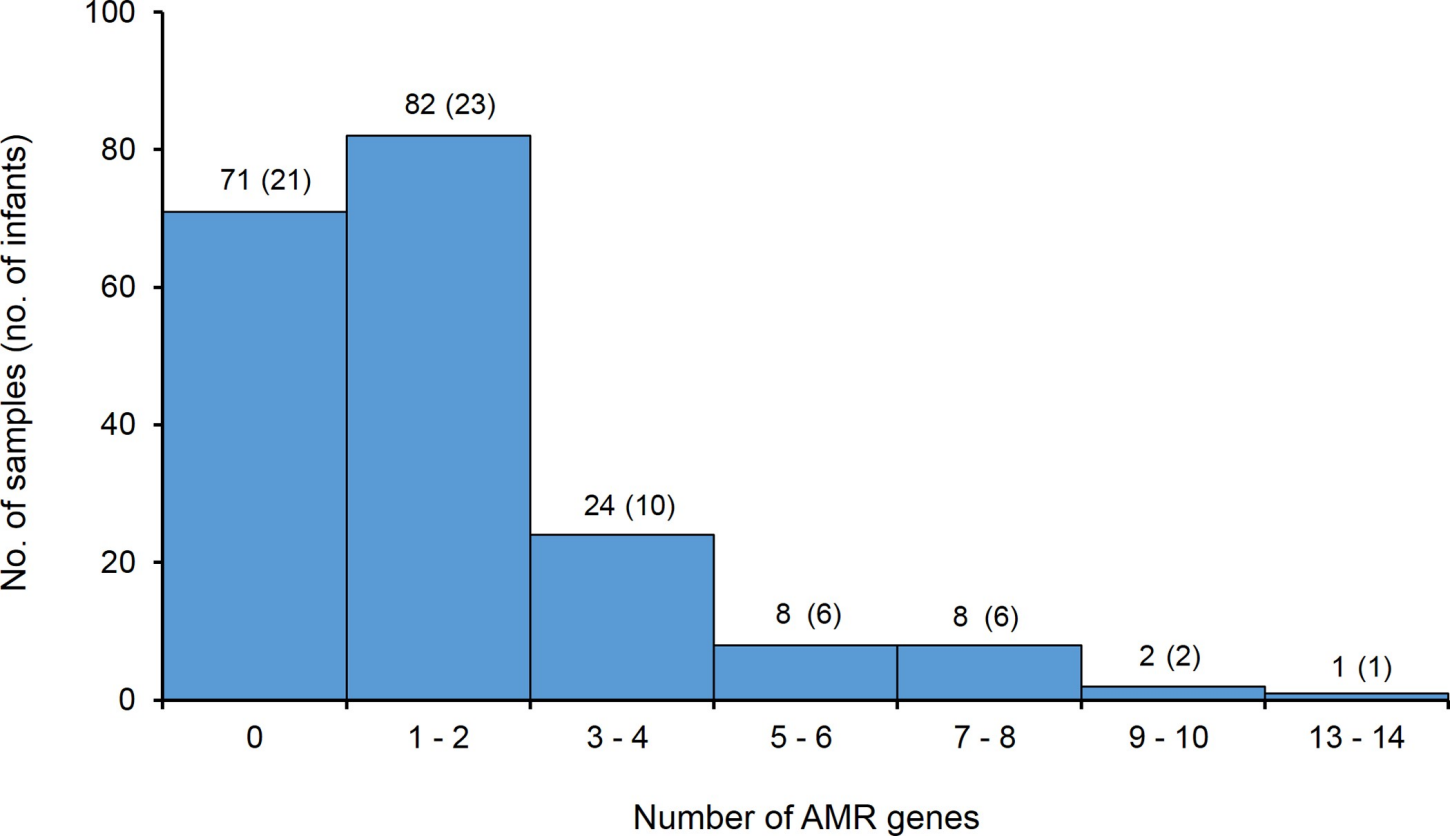
Distribution of antimicrobial-resistance (AMR) genes within 196 longitudinal NP samples selected from 23 infants.

**Fig 2:**
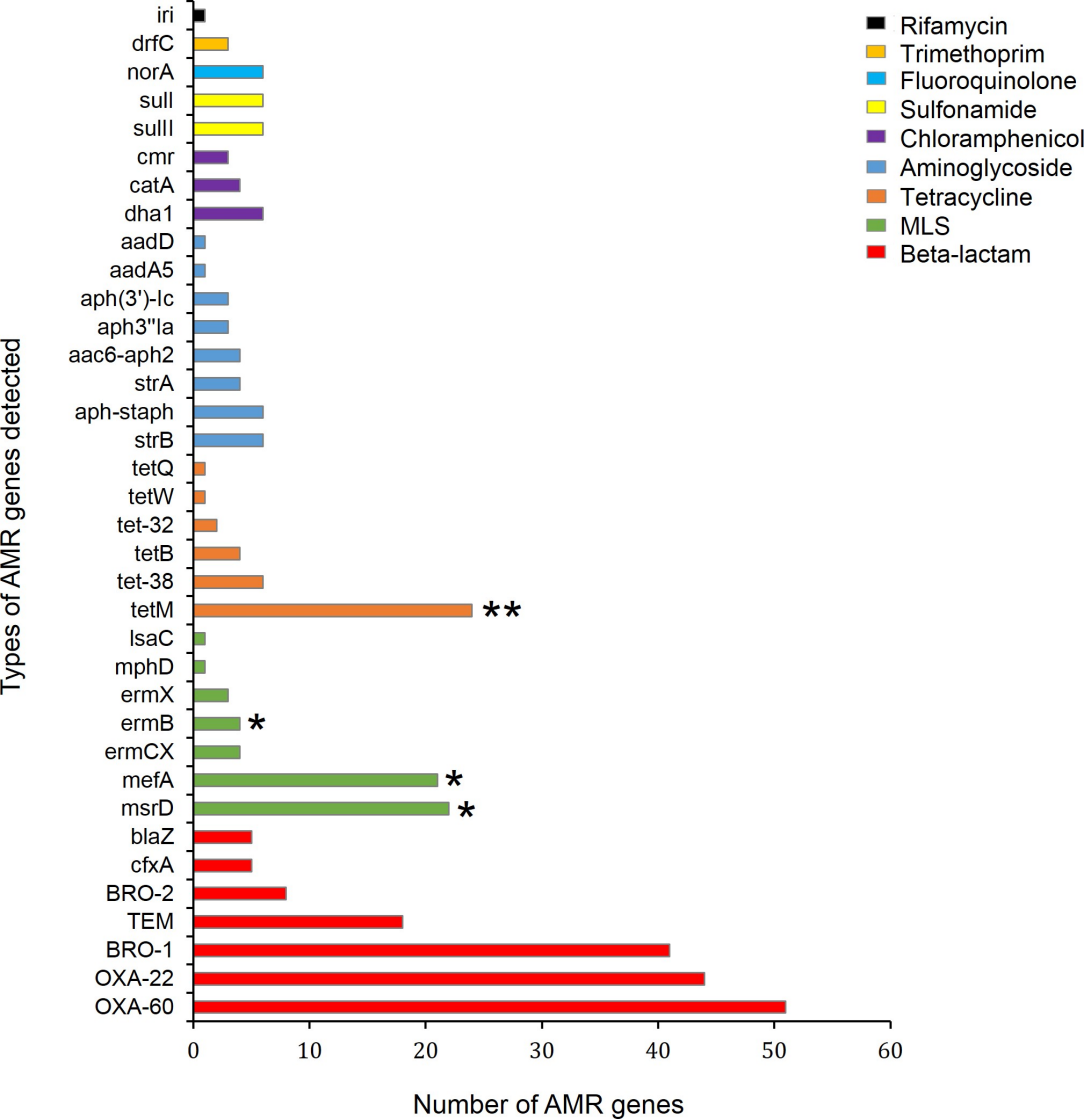
Frequency of antimicrobial-resistance (AMR) genes within 196 longitudinal NP samples selected from 23 infants. MLS-Macrolide-lincosamide-streptogramin. (*) AMR genes detected from streptococcal contigs. (**) AMR gene detected from streptococcal contigs in 23 out of 24 samples.

**Fig 3:**
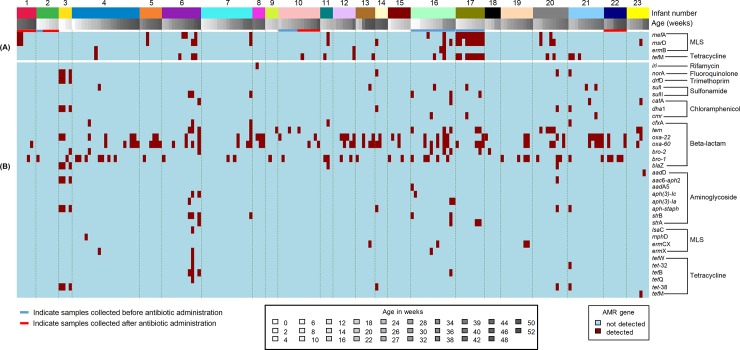
Antibiotic-resistance genes detected from 196 longitudinal NP samples selected from 23 infants (assigned 1 to 23). (A) Streptococcal resistome. (B) Non-streptococcal resistome. Blue line indicates samples collected before antibiotic administration, for lower respiratory tract infection. Red line indicates samples collected after antibiotic administration, for lower respiratory tract infection. MLS- Macrolide-lincosamide-streptogramin, AMR- Antimicrobial resistance.

The average depth of coverage of the detected AMR genes from all contigs was 26X (range 1 – 862X). A total of 329 AMR genes were detected in 64% (125/196) of the selected NP samples. Among these, 57% (188/329) were detected at ≥ 90% identity and ≥ 25% gene coverage while only 30% (97/329) were detected at the more stringent cut-off of ≥ 90% identity and ≥ 80% gene coverage (Table 2). The number of resistance genes detected per sample ranged from 1–14 genes (Fig 1), and included 36 non-redundant genes (Fig 2). AMR genes were detected in at least one sample from each of the 23 selected infants (Fig 3). The same types and number of AMR genes were detected from all bacterial contigs combined compared to those detected from non-streptococcal and streptococcal contigs separately. The most common resistance genes detected were those conferring resistance to beta-lactams (52%, 172/329), macrolide-lincosamide-streptogramin antibiotics (MLS) (17%, 56/329), and tetracyclines (12%, 38/329) (Table 2 and Fig 2). A high number of AMR genes conferring resistance to MLS (n = 38), tetracyclines (n = 25), aminoglycosides (n = 17), fluoroquinolones (n = 4), and trimethoprim (n = 3) were detected at a cut-off of 90% identity over 50% gene coverage (Table 2). Different patterns of carriage of AMR genes were observed, with only one infant having a stable carriage of *mef*A, *msr*D and *tet*M over a long period (Fig 3).

**Table 2:** Detection of AMR genes conferring resistance to various classes of antibiotics from 196 NP samples using different stringency criteria.

Antibiotic class	Number of genes			Total number (%)
	≥ 90% ID	≥ 90% ID	≥ 90% ID	
	≥ 25% Cov	≥ 50% Cov	≥ 80% Cov	
Bla	125	29	18	172 (52.3)
MLS	18	4	34	56 (17.0)
Tet	13	2	23	38 (11.6)
AGly	11	8	9	28 (8.5)
Phe	8	0	5	13 (4.0)
Sul	10	1	1	12 (3.6)
Flq	2	0	4	6 (1.8)
Tmt	0	0	3	3 (0.9)
Rif	1	0	0	1 (0.3)
Total	188	44	97	329 (100.0)

AMR- Antimicrobial-resistance, Bla- Beta-lactam, MLS- Macrolide-lincosamide-streptogramin, Tet- Tetracycline, AGly- Aminoglycoside, Phe- Chloramphenicol, Sul- Sulfonamide, Flq- Fluoroquinolone, Tmt- Trimethoprim, Rif- Rifamycin, ID- identity, Cov- coverage.

### Streptococcal resistome

Shotgun sequencing detected streptococcal reads in all 174 samples that were culture positive for *S*. *pneumoniae*. Seventy AMR genes (four non-redundant genes) were detected from streptococcal contigs; the average depth of coverage was 103X (range 1 – 411X). MLS and tetracycline resistance genes were the only genes detected from streptococcal contigs. The most commonly detected gene was *tet*M (n = 23), followed by *msr*D (n = 22), *mef*A (n = 21), and *erm*B (n = 4). *Msr*D, *erm*B, and *mef*A genes were only identified from streptococcal contigs. The combination of *msr*D, *mef*A and *tet*M genes was detected in 10 samples from 3 infants and all were identified on the same contig in 9 out of 10 samples.

### Non-streptococcal resistome

A total of 259 AMR genes were detected from non-streptococcal contigs with an average coverage depth of 10X (range, 1 – 862X). Nine types of AMR genes were detected from non-streptococcal contigs (Fig 3), with beta-lactam resistance genes (66%, 172/259) being the most commonly detected genes (Table 2 and Fig 2). The most commonly detected beta-lactam resistance gene was *bla*_OXA-60_, followed by *bla*_OXA-22_, *bla*_BRO-1_, *bla*_TEM_, *bla*_BRO-2_, *cfx*A, and *bla*Z (Fig 2). The fluoroquinolone resistance gene *nor*A was detected in six samples from four infants (Fig 3).

### Association between antibiotic use and the NP resistome

No significant difference was observed between the presence of AMR genes in samples collected before and after the treatment of LRTI (Fig 3). A large proportion of AMR genes (69% 227/329) were detected in samples from a subset of eight infants (Fig 3).

## Discussion

This proof-of-concept study investigated the composition of the NP antibiotic resistome in an intensively sampled South African birth cohort. 329 AMR genes were detected across 64% of the selected NP samples using targeted enrichment culture and shotgun metagenomic sequencing. We detected the same types and number of AMR genes from all contigs combined compared to those detected from non-streptococcal and streptococcal contigs separately, suggesting that our resistome analysis was reliable. The average depth of coverage for the resistance genes from streptococcal contigs (103X) was higher than that from non- streptococcal contigs (10X). This observation is likely due to the short streptococcal enrichment culture step using Todd Hewitt broth (without antibiotics) [24].

We detected resistance genes using a lower stringency criteria of ≥ 90% identity over 25% coverage of the reference gene, which has been shown to be more reliable, than more stringent criteria, in detecting AMR genes [27]. Yang *et al*., reported a high accuracy (99%) for detecting AMR genes using these less stringent parameters in metagenomic analysis [28]. In the current study, at least 25% gene coverage was used [26], and this cut-off was higher than the suggested coverage of ≥ 25 amino acids [28]. Only 30% of the AMR genes were detected using ≥ 90% identity over 80% coverage of the reference gene [26]. The more stringent parameters detected mainly MLS, tetracycline, and aminoglycoside resistance genes which are frequently carried by *Streptococcus* species, presumably due to the higher depth of coverage as a result of the enrichment step [29,30].

We observed differences in the types and numbers of AMR genes identified from streptococcal and non-streptococcal contigs. With the exception of one sample in which *tet*M was detected; *tet*M, *msr*D, *erm*B, and *mef*A genes were only detected from streptococcal contigs (Fig 3). The *msr*D, *erm*B, and *mef*A genes are most frequently detected among streptococcal isolates [31]. Pneumococci which are resistant to MLS antibiotics are commonly also resistant to tetracycline due to the insertion of an MLS gene into the conjugative transposons of the Tn*916* family, which typically carry the *tetM* gene [32,33]. Although transposons were not evaluated in the current study, *msr*D and *tet*M genes were commonly identified on the same contig (9/10 samples) suggesting they could be carried on the same transposon [33].

The predominant AMR genes detected from non-streptococcal contigs were beta-lactamase genes, specifically *bla*_OXA-60_, *bla*_OXA-22_, *bla*_BRO-1_, and *bla*_TEM_. All *bla*_TEM_ gene variants detected in the current study encode narrow spectrum beta-lactamase enzymes, and these have previously been detected in the *Enterobacteriaceae*, *H*. *influenzae*, and *Neisseria gonorrhoea* [34]. The *bla*_BRO-1_ gene was more commonly detected than *bla*_BRO-2_ (Fig 2), both are typically found among *Moraxella catarrhalis* isolates, with *bla*_BRO-1_ more prevalent than *bla*_BRO-2_ in *M*. *catarrhalis* [35].

*bla*_OXA-60_ and *bla*_OXA-22_ genes, encoding the chromosomal and inducible class D beta-lactamases have only been described in *Ralstonia pickettii* or *R*. *mannitolilytica* [36–40]. *Ralstonia* sp. are Gram-negative, non-fermentative bacilli, commonly isolated from the respiratory tract and their carriage among infants in this study warrants further investigation [41]. OXA-22 is an oxacillinase with the ability to hydrolyse narrow-spectrum beta-lactams [40]. Unlike OXA-22, the hydrolysis spectrum of OXA-60, although narrow, includes carbapenems. Whilst *R*. *pickettii* infrequently causes infections, the potential for transfer of this gene to other NP bacteria should be studied [36,39].

The *nor*A gene, which encodes a fluoroquinolone efflux transporter protein, has been described mainly in *Staphylococcus aureus* and can render resistance to both fluoroquinolones and other classes of antibiotics with dissimilar structures [42,43].

Beta-lactamase genes were the most commonly detected resistance genes in the current study. Amoxicillin, a beta-lactam antibiotic, was the most commonly prescribed antibiotic for both acute and ambulatory care in children in this study, which could explain the high number and types of beta-lactamase genes detected [44].

There were several limitations to the current study. Firstly, the enrichment culture for streptococci altered the composition of the NP resistome and the prevalence of the different AMR genes detected may therefore differ from that found in directly tested samples. Secondly, the purposively selected sample set is unlikely to be broadly representative of infants in this study. Thirdly, the reference database used for the resistome analysis is not comprehensive, and excludes chromosomal mutations associated with resistance. Penicillin resistance associated with *pbp* gene mutations, such as *pbp*1a and *pbp*2x, and trimethoprim sulphamethoxazole resistance, associated with *fol*A I100L substitutions and *fol*P insertions, would not be detected using this database, and further work will be done to characterise these associations.

This study demonstrates that WMGS can provide a broad snapshot of the NP resistome. Recent work has highlighted that the nasopharynx is a conducive environment for the exchange of AMR genes between related *Streptococcus* species responsible for respiratory tract infections in children [45]. WMGS has the potential to provide a comprehensive assessment of all resistance elements present in this niche.
